# Supplementary figures and images for: Quantitative proteomics of cerebrospinal fluid from African Americans and Caucasians reveals shared and divergent changes in Alzheimer’s disease
Source: Mol Neurodegener. 2023 Jul 19;18:48. doi: 10.1186/s13024-023-00638-z (PMC10355042; doi:10.1186/s13024-023-00638-z)

## Supplemental Figure 1

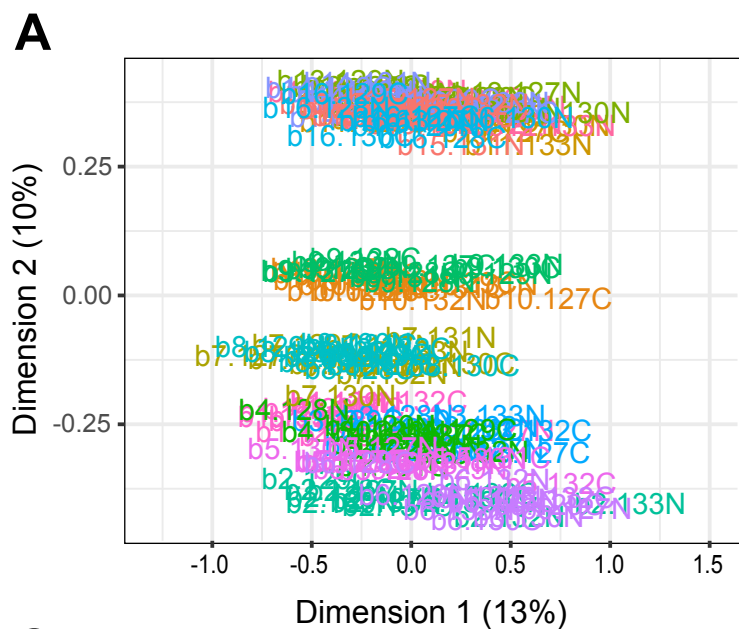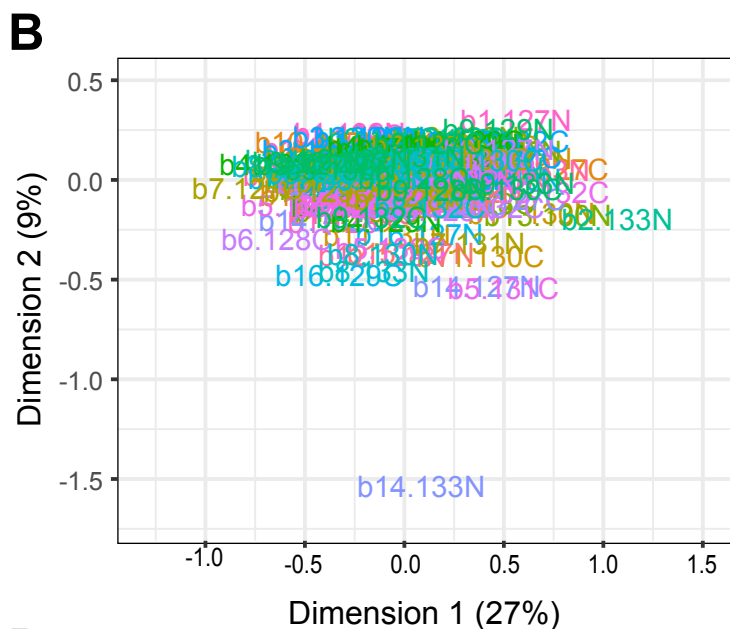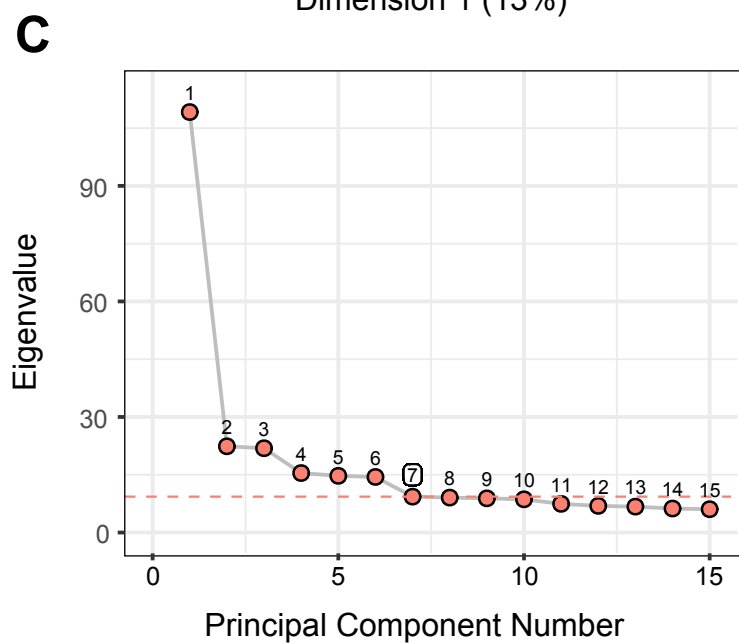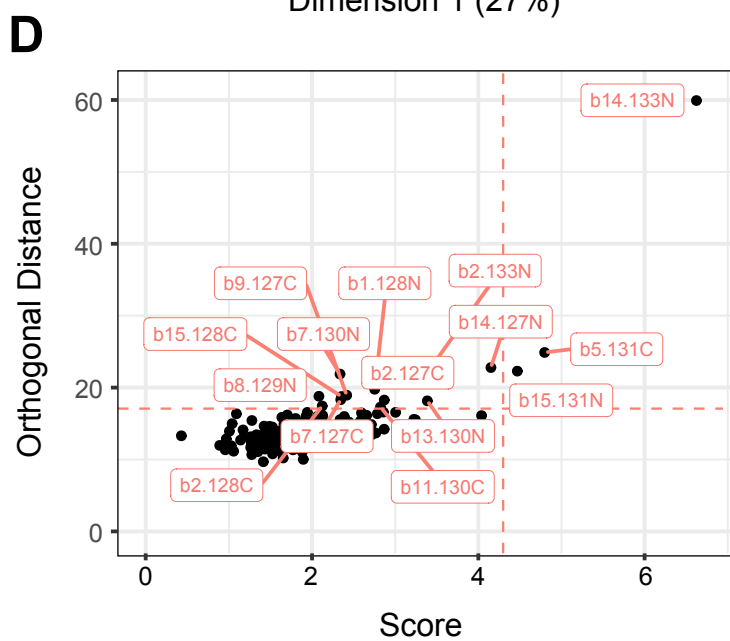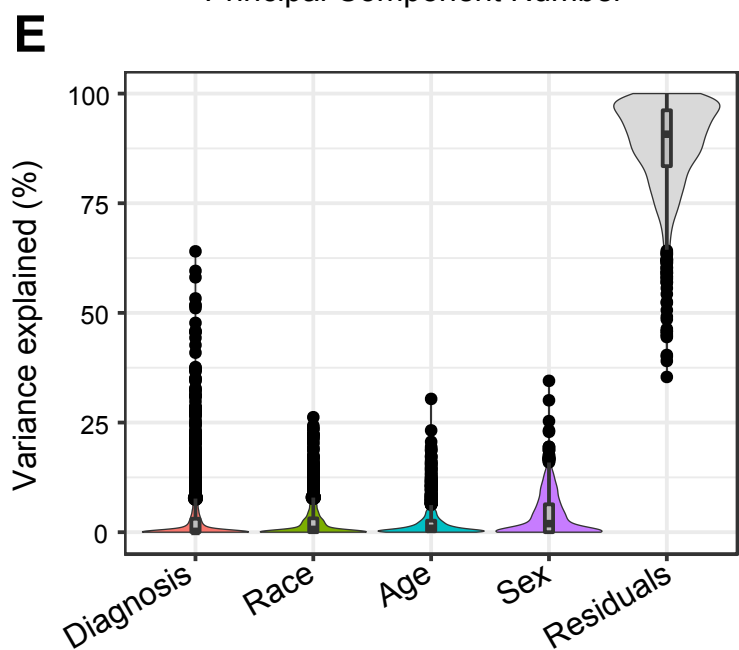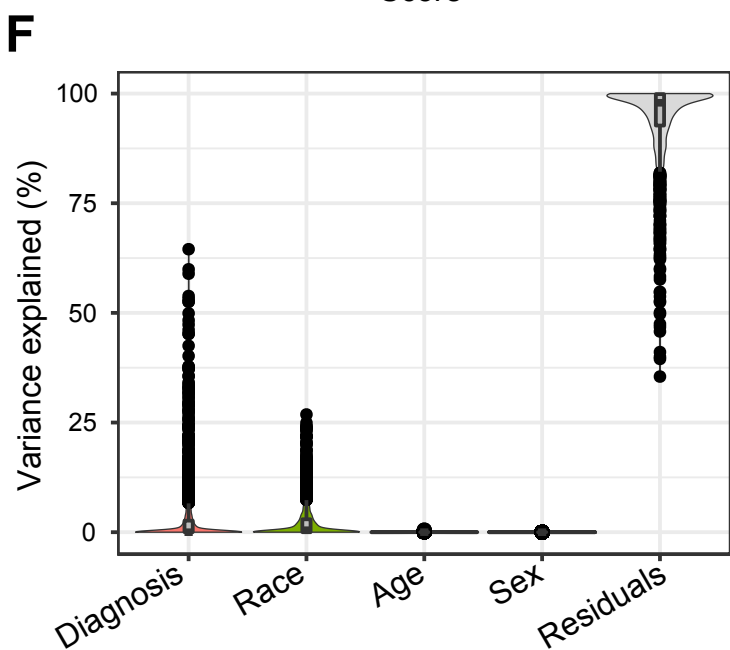

Supplement: Supplementary file 2 — Additional file 2: Supplemental Figure 1. Batch correction, outlier removal and bootstrap regression. Multidimensional scaling (MDS) plots were used to illustrate batch contributions to variance before and after batch correction. In MDS plots, the distance a case is from one another is reflective of how similar or dissimilar a case is from the other. (A) Prior to batch correction, the samples clustered by batch (B) After batch correction, the samples no longer cluster by batch. (C) After batch correction, a principal component (PC)-based outlier removal method was utilized to detect outliers. By graphing the eigenvalue of each component against the PC number, the elbow or bend in the graph, which in this case was 7, was indicative of the ideal number of components to include within the parameters. (D) With a criterion for computing cutoff values set to 0.99, the cutoffs for the detection of outliers for the orthogonal distance and score were 16.79257 and 4.654674 respectively. This resulted in the detection of 15 outliers (b1.128N, b11.130C, b13.130N, b14.127N, b14.133N, b15.128C, b15.131N, b2.127C, b2.128C, b2.133N, b5.131C, b7.127C, b7.130N, b8.129N, b9.127C). B14.133N was such an extreme outlier because it was an empty channel. (E) After outlier removal, the matrix underwent bootstrap regression to remove variations in the dataset that were due to age and sex. Variance partition plots were employed to illustrate the percent contribution of diagnosis, race, age, and sex to the variance of each protein. (F) Following bootstrap regression, variations explained by age and sex were removed. [file 13024_2023_638_MOESM2_ESM.pdf]

**A**

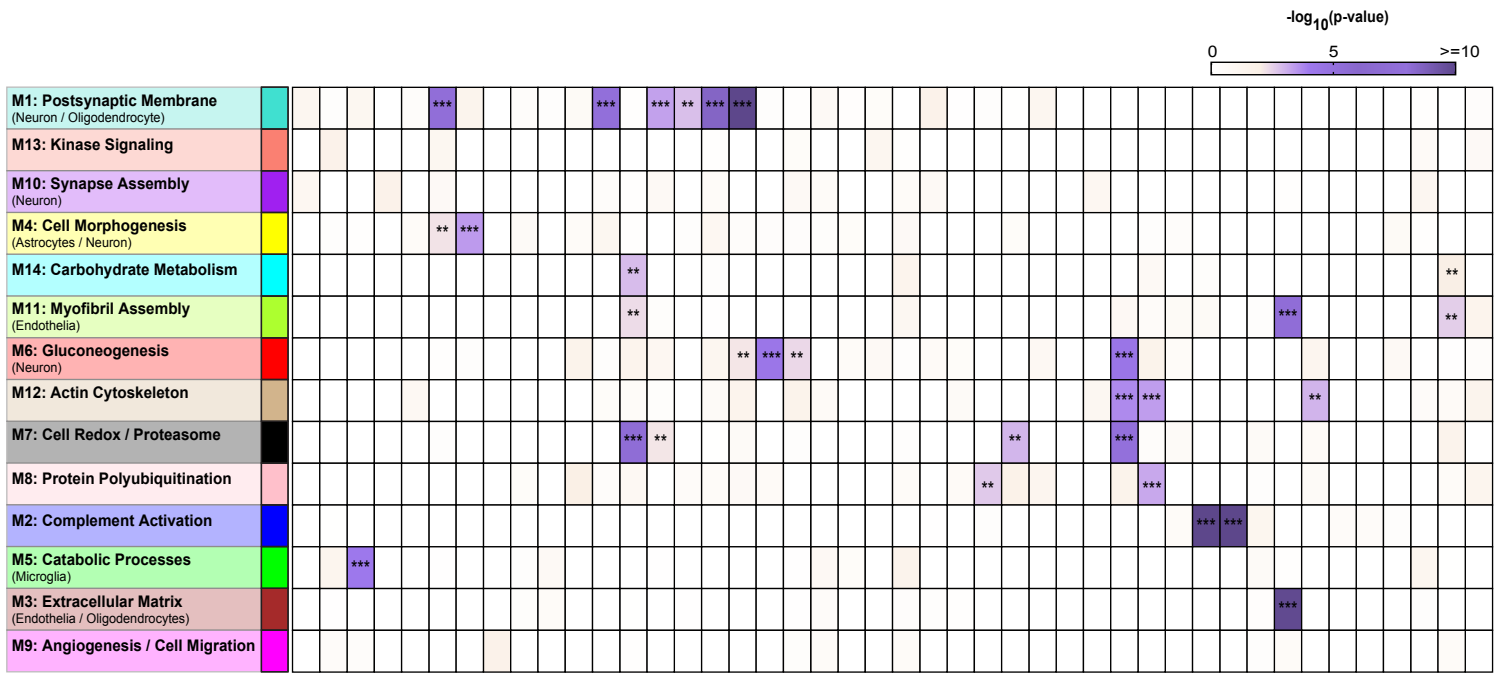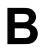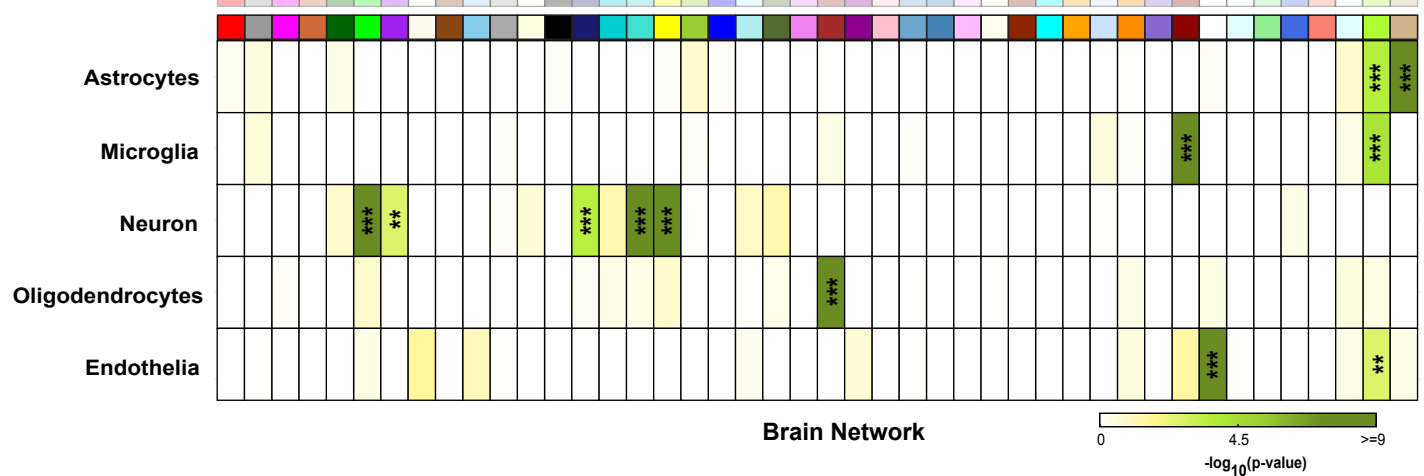

Supplement: Supplementary file 3 — Additional file 3: Supplemental Figure 2. (A)Protein module enrichment across the CSF and brain was assessed by matching gene symbols of proteins in each module from the CSF network against gene symbols for protein in each module from a human AD consensus brain network using a one-tailed Fisher’s exact test. The degree of enrichment increases from pink to light purple to dark purple with asterisks denoting the following statistical significance (**p≤0.01 and ***p≤.001). (B) Similar to CSF, cell-type enrichment was assessed by cross referencing brain module proteins against a list of proteins determined to be enriched in neurons, oligodendrocytes, astrocytes, and microglia using a one-tailed Fisher’s exact test. The degree of cell-type enrichment increases from yellow to green-yellow to dark green with asterisks denoting the following statistical significance(**p≤0.01 and ***p≤.001). [file 13024_2023_638_MOESM3_ESM.pdf]

A

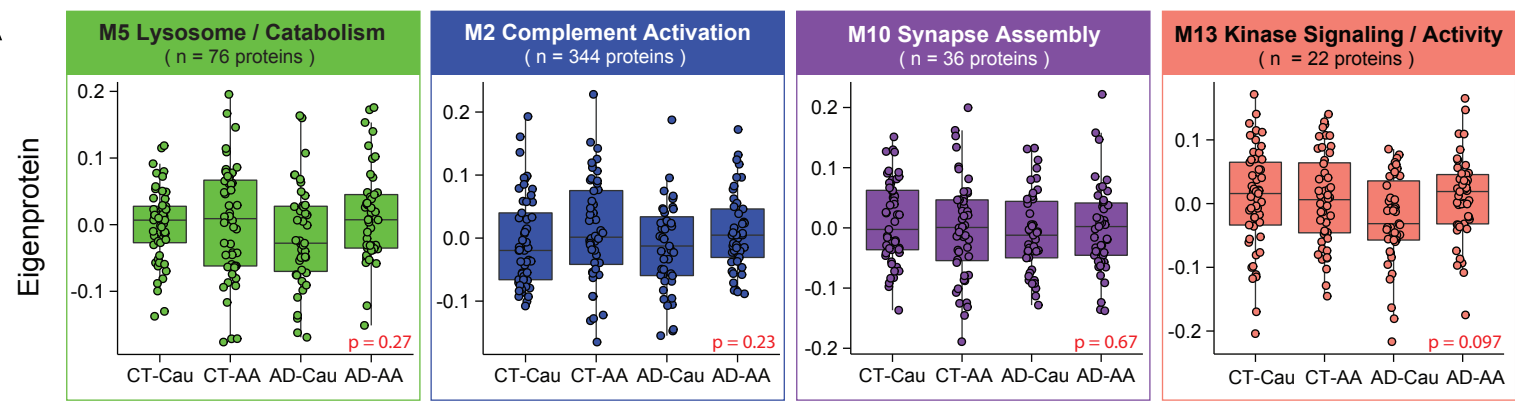

Supplement: Supplementary file 4 — Additional file 4: Supplemental Figure 3. Additional CSF network protein modules. (A) Eigenprotein levels were distributed by race and diagnosis for remaining modules not shown in main Figure 4. This includes M5, M2, M10, and M13. [file 13024_2023_638_MOESM4_ESM.pdf]

# Supplemental Figure 4

**A**

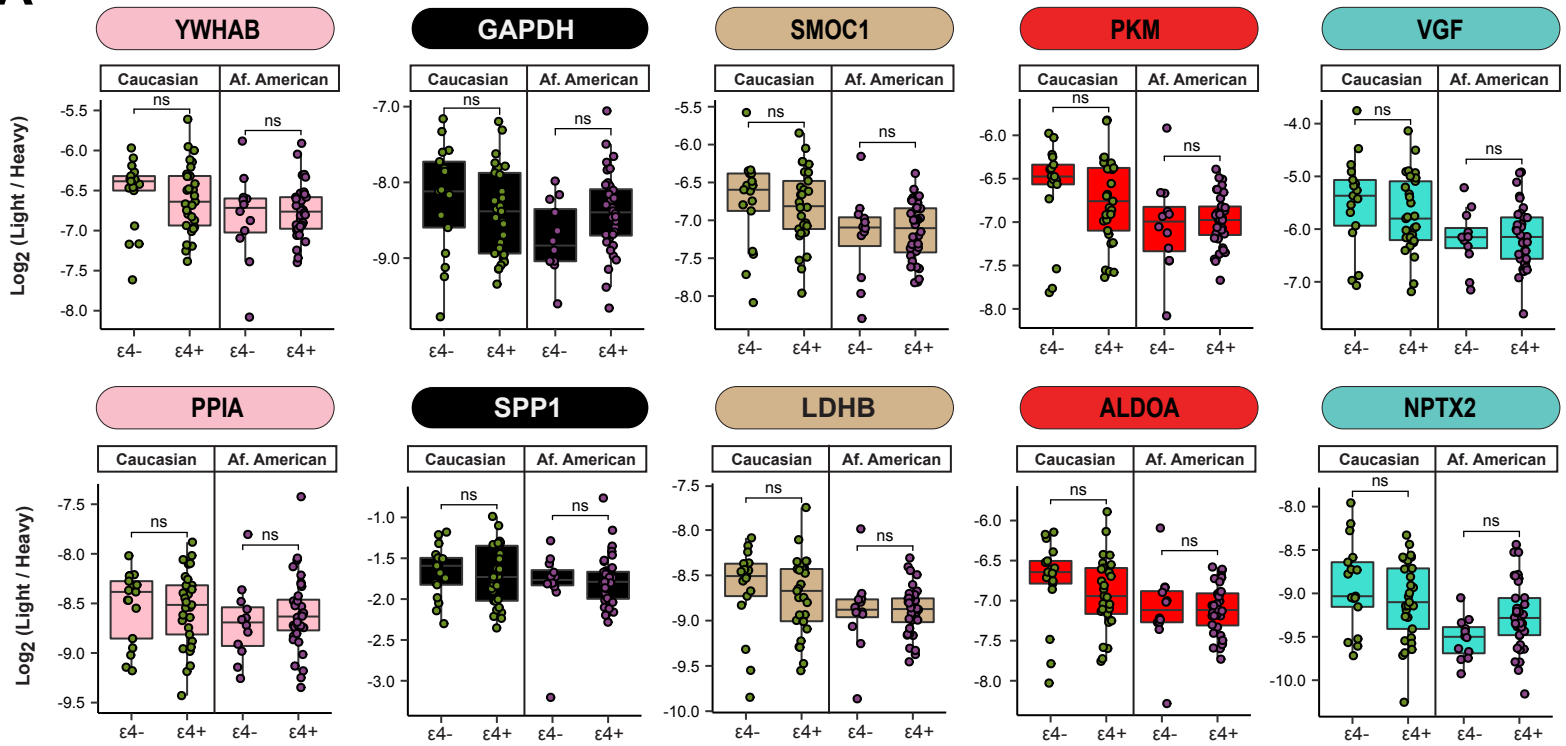

**B**

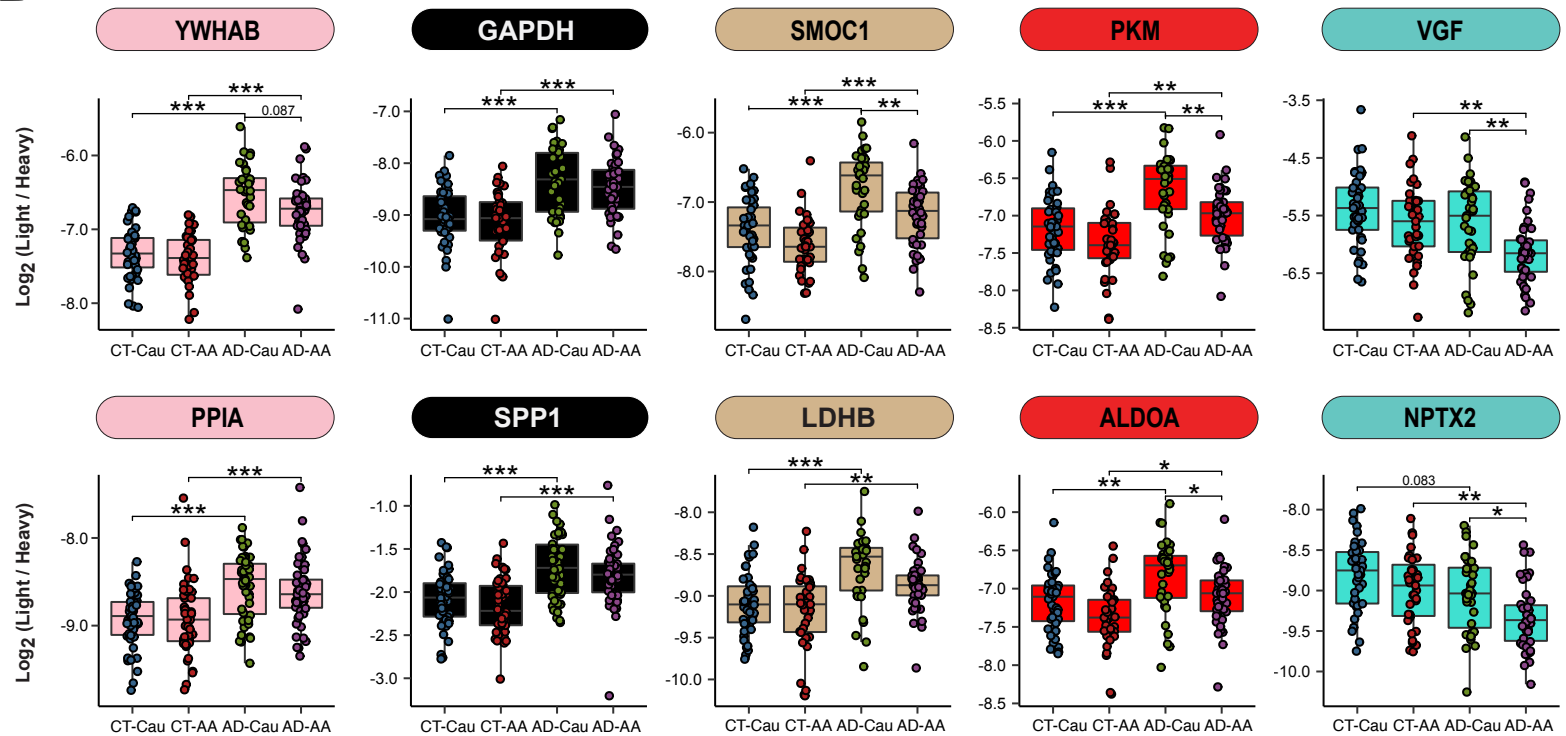

Supplement: Supplementary file 5 — Additional file 5: Supplemental Figure 4. Stratification of SRM CSF protein measurements in by APOE genotype and comorbidity. (A) Within each race, protein levels for were not affected by APOE ε4 genotype for YWHAB, GAPDH, SMOC1, PKM, VGF, PPIA, SPP1, LDHB, ALDOA, and NPTX2. (B) Within each race, protein levels for were not affected by patient co-morbidities (hypertension, diabetes, dyslipidemia, or cerebrovascular disease) for YWHAB, GAPDH, SMOC1, PKM, VGF, PPIA, SPP1, LDHB, ALDOA, and NPTX2. Only cases with co-morbidities (Supplemental Table 1) were included in these boxplots. [file 13024_2023_638_MOESM5_ESM.pdf]
